# Piloting the Elder Abuse Suspicion Index – long‐term care (EASI‐ltc©): A Mixed Methods Feasibility Study

**DOI:** 10.1002/alz.089643

**Published:** 2025-01-09

**Authors:** Machelle Wilchesky, Mélanie Couture, Mark J Yaffe, Stephanie A. Ballard, Maryse Soulières, Sarita Israel, Ilanah Milgram

**Affiliations:** ^1^ McGill University, Department of Family Medicine, Montreal, QC Canada; ^2^ Centre for Research in Aging ‐ Donald Berman Maimonides Geriatric Centre, Montreal, QC Canada; ^3^ Université de Sherbrooke, Sherbrooke, QC Canada; ^4^ Université de Montréal, Montreal, QC Canada; ^5^ CIUSSS du Centre‐Ouest‐de‐l'île‐de‐Montréal, Montreal, QC Canada

## Abstract

**Background:**

Long‐term care (LTC) residents are a previously untested and highly vulnerable population at risk of elder abuse (EA) and its many negative health outcomes. The detection of elder abuse within the LTC context is urgent and time‐sensitive.

**Objective:**

The overarching aim of this study is to evaluate the feasibility of implementing the Elder Abuse Suspicion Index – long‐term care (EASI‐ltc^©^): the first comprehensive detection tool of its kind designed specifically to identify the abuse of cognitively‐apt persons living in LTC. Preliminary tool validity will also be evaluated.

**Methods:**

This observational pilot study is taking place within seven LTC institutions in Montreal Canada. It begins with the administration of the EASI‐ltc on a random sample of eligible LTC residents. Residents subsequently undergo a follow‐up assessment led by a trained and experienced social worker, which is used as the ‘silver standard’ reference for validation. Residents are asked to reflect on their own experiences as participants in one on‐one interviews, and key stakeholders (e.g., administrators, staff members, family, companions, and friends) are asked to provide retroactive feedback via an online survey. Survey respondents are also invited to participate in interviews to clarify their responses. Potential harmful consequences arising from EASI‐ltc administration is being evaluated.

**Results:**

Results pertaining to tool validity, feasibility, and acceptability obtained from data collected during the first 8 months will be presented.

**Conclusion:**

The EASI‐ltc is the first published comprehensive tool to detect elder abuse in this vulnerable population. This ongoing study responds to an urgent need to provide tools to identify abuse and give a voice to LTC residents locally, nationally, and beyond.

